# Nutrition in children with CRF and on dialysis

**DOI:** 10.1007/s00467-006-0279-z

**Published:** 2007-10-01

**Authors:** Lesley Rees, Vanessa Shaw

**Affiliations:** 1 Department of Nephrourology, Gt Ormond St Hospital for Children NHS Trust, Gt Ormond St, London, WC1N 3JH UK; 2 Department of Dietetics, Gt Ormond St Hospital for Children NHS Trust, Gt Ormond St, London, WC1N 3JH UK

**Keywords:** Nutrition, Chronic renal failure, Dialysis, Growth

## Abstract

The objectives of this study are: (1) to understand the importance of nutrition in normal growth; (2) to review the methods of assessing nutritional status; (3) to review the dietary requirements of normal children throughout childhood, including protein, energy, vitamins and minerals; (4) to review recommendations for the nutritional requirements of children with chronic renal failure (CRF) and on dialysis; (5) to review reports of spontaneous nutritional intake in children with CRF and on dialysis; (6) to review the epidemiology of nutritional disturbances in renal disease, including height, weight and body composition; (7) to review the pathological mechanisms underlying poor appetite, abnormal metabolic rate and endocrine disturbances in renal disease; (8) to review the evidence for the benefit of dietetic input, dietary supplementation, nasogastric and gastrostomy feeds and intradialytic nutrition; (9) to review the effect of dialysis adequacy on nutrition; (10) to review the effect of nutrition on outcome.

## The importance of nutrition in normal growth

Normal growth can be divided into four important phases: prenatal, infantile, childhood and pubertal. Nutrition is important at all phases of growth, but particularly so during the infantile phase because the rate of growth is higher than at any other time of life (other than prenatally) and is less dependent on growth hormone (GH) than during other phases. Rate of growth gradually decreases from >25 cm/year at birth to an average of 18 cm/year at age 1 year and 10 cm/year by the age of 2. Half of adult height is achieved by the age of 2 years, so that irrecoverable loss of growth potential can occur during this phase. At birth, 170 kcal/day are stored in new tissue, falling to 50–60 at 6 months, 30–40 by 1 year and 20–30 by the age of 2 years. During the childhood phase, growth becomes more dependent on the GH/insulin-like growth factor-1 (IGF-1) axis; growth rate decelerates continuously until the pubertal phase. The pubertal phase results from the coordination of GH and sex steroid production. Together they have an anabolic effect on muscle mass, bone mineralization and body proportions. It is another phase of rapid growth so that nutrition can again modify the genetic growth potential [1].

## Methods of assessing nutritional status

Normal nutrition can be defined as maintenance of normal growth and body composition. Although it is agreed that nutritional assessment is important in chronic renal failure (CRF), there is no single or easy definition or measure of inadequate nutritional status: measurement of nutritional parameters are complicated in CRF because of salt and water imbalances and the potential inappropriateness of using age matched controls in a population that is short and may be delayed in puberty; it has been suggested that it is more appropriate, therefore, to express measures relative to height age (age at which the child’s height would be the 50th centile) and/or pubertal stage. The reader is referred to extensive and excellent reviews on this subject [2, 3].

### Anthropometric measures

The most commonly used assessment of nutrition is height and weight, along with head circumference in younger children, plotted on percentile charts. Anthropometric and nutritional measures are usefully expressed as a score of the number of standard deviations (SDs) from the mean for a normal population of the same age [e.g. height or weight SD score (HtSDS, WtSDS), also called z scores]. This allows comparison with the normal population and helps follow progress in the individual patient. However, although a normal rate of growth can be considered to represent adequate nutrition, weight loss and alterations in body composition occur before height velocity is affected [2], and poor growth can occur due to reasons other than nutrition (see below).

Another way of expressing the relative weight and height is the body mass index (BMI, Ht/Wt^2^), which is important because extremes are associated with increased morbidity and mortality [4]. Because BMI varies considerably throughout childhood, reaching a trough at 4–6 years of age, it has been suggested that it should be calculated according to height age [5]. It must be borne in mind that BMI does not distinguish between fat mass and fat-free mass (FFM) and an appropriate BMI for age (whether height age or chronological age) does not necessarily indicate ideal body composition; weight gain may be due to the laying down of excess fat rather than a balance of fat and lean tissue.

Skinfold thickness is a measure of subcutaneous fat and mid-arm circumference (MAC) is a reflection of muscle mass and may therefore be more useful in determining body composition than the calculation of BMI alone. Decreased values have been found in children with CRF [6–9]. However, both are rather unreliable tools because consistent measurements are difficult, values may not be representative of visceral fat and fat or muscle mass, oedema will influence values, regional fat and muscle distribution may be different in CRF and values vary according to age in normal children.

### Dietary assessment

The paediatric renal dietician is crucial to the successful management of nutrition in children with renal disease. Monthly review has been recommended for under twos on dialysis, and three- to four-monthly in those over that age [10]; and six-monthly or one- to three monthly in children with moderate and severe CRF, respectively [3]. The purpose is to prevent the development of malnutrition. Children entirely dependent on enteral feeds may need to be seen more often, particularly in infancy when feed adjustments may be necessary as often as weekly. Assessment of intake in children taking an oral diet can depend on prospective dietary diaries, usually over 3 days, or retrospective recall. It has been estimated that 5.9 contacts per patient (in clinic or by phone) per month in children <5 years of age and 3.1 in children >5 years of age are necessary to successfully support families of children on peritoneal dialysis (PD). This intensive input resulted in improvement of HtSDS and WtSDS from −1.2 and −1.32 to −1.14 and −0.73, respectively, over a 3-year period [11]. Protein intake can also be calculated using well established formulae (protein catabolic rate, nPCR [2]).

### Serum albumin

Serum albumin has been identified as a surrogate marker for nutritional status and morbidity/mortality in patients with end-stage renal failure (ESRF). Patients <18 years of age initiating dialysis with hypoalbuminemia are at a higher risk for death: in 1,723 children each fall of serum albumin by 1 g/dl at the start of dialysis was associated with a 54% higher risk of death. This was independent of other potential confounding variables [12].

Although serum albumin may be a reflection of nutrition, low levels may be due to haemodilution, nephrotic syndrome or chronic infection/inflammation [13]. To remove the effect of fluid overload, therefore, it has been suggested that if practical, levels should be checked post dialysis [14]. Chronic inflammation in itself will lead to malnutrition [15]. Low serum albumin is more common in children on PD: low levels were present in 35.9% of assays in 39 children on PD over a 2-year period compared with none in 32 children on haemodialysis (HD), even though protein intake (estimated by nPCR) was similar, averaging 1.1 g/kg per day. Thus, children maintained on PD are at greater risk of protein malnutrition compared with peers treated with HD [16]. This may be due in part to losses in PD fluid: average losses of free amino acids (AA) vary with transporter status from 0.02 to 0.03 g/kg/day [17]. There is an inverse correlation between body weight and surface area and peritoneal protein loss, such that infants have nearly twofold greater peritoneal protein losses per metre-square body surface area than those weighing more than 50 kg. Such protein losses in infants impair normal growth and may contribute to permanent loss of growth potential [18].

### Dual energy X-ray absorptiometry (DEXA) and other methods

A whole body DEXA scan can estimate fat mass, lean mass and bone mineral density (BMD), but is affected by body water content. Other methods include bioelectrical impedance analysis (BIA) [19], total body potassium, densitometry and in vivo neutron activation analysis, but these are predominantly used as research tools [2].

## Nutritional requirements for normal children

Recommended daily amounts (RDA) and recommended intakes (RI) for energy, protein and nutrients vary between countries. Regardless of the national dietary recommendations that are used it is important to consider that these are estimates of requirements for normal healthy populations of people and are not recommendations for absolute intakes for individuals. They serve as a guide for the energy and nutrients that an individual may require for normal growth, maintenance, development and activity. Requirements for any particular nutrient will differ between individuals.

The United Kingdom dietary reference values (DRV) [20] are for infants fed artificial formulas and for older infants, children and adults consuming food. DRVs are not set for breast-fed babies, as it is considered that human milk provides the necessary amounts of nutrients. In some cases the DRVs for infants aged up to 3 months who are formula-fed are in excess of those which would be expected to derive from breast milk; this is because of the different bioavailability of some nutrients from breast and artificial milk.

The DRV for energy intake is assumed to be normally distributed and is expressed as the estimated average requirement (EAR). For protein and other nutrients, requirements are expressed as reference nutrient intakes (RNI), set at 2 SDs above the average. Therefore, intakes of protein and nutrients above this amount will almost certainly be adequate for all individuals in a population. For some nutrients where there is insufficient data to establish DRVs with great confidence safe intakes are set—a level or range of intake at which there is no risk of deficiency.

Daily DRVs for energy, protein and some nutrients are given in Table 1. If a diet for a normal child is adequate in calcium, iron and vitamin C and the child is receiving adequate energy from a mixed diet, then most other nutrients are likely to be taken in adequate amounts.

**Table 1:** UK dietary reference values for normal populations of children [20] (*mo* months, *yr* years)

Age	EAR	RNI	RNI	RNI	RNI
	Energy (kcal)	Protein (g)	Calcium (mmol)	Iron (mg)	Vitamin C (mg)
0–3 mo	115–100/kg	2.1/kg	13.1	1.7	25
4–6 mo	95/kg	1.6/kg	13.1	4.3	25
7–9 mo	95/kg	1.5/kg	13.1	7.8	25
10–12 mo	95/kg	1.5/kg	13.1	7.8	25
1–3 yr	95/kg	1.1/kg	8.8	6.9	30
4–6 yr	90/kg	1.1/kg	11.3	6.1	30
7–10 yr	1,970/day	28.3/day	13.8	8.7	30
Boy 11–14 yr	2,220/day	42.1/day	25.0	11.3	35
Girl 11–14 yr	1,845/day	41.2/day	20.0	14.8	35
Boy 15–18 yr	2,755/day	55.2/day	25.0	11.3	40
Girl 15–18 yr	2,110/day	45.0/day	20.0	14.8	40

## Nutritional requirements for children with CRF, on dialysis and post transplant

### Energy

It is unlikely that energy requirements for children with CRF are different from those of normal children, and energy intakes below the EAR will contribute to growth failure. Restoration of normal energy requirements to 100% EAR allows for catch-up growth in children under 2 years and shows some benefit in older children (see section on Dietary supplements). If recurrent vomiting resulting from abnormal gastric motility and delayed gastric emptying is not treated, energy intake may need to be increased by up to 30% daily to replace lost feed and food in order to preserve growth; once vomiting is controlled growth is maintained on normal energy requirements [21]. To ensure an adequate energy intake it is advisable to use the EAR for height age if the child is below the 2nd centile for height.

There is no evidence that intakes for children on dialysis should exceed those for normal children [10], though dietary energy intake may need to be reduced for children on PD to compensate for the energy derived from dialysate glucose, estimated at 8–12 kcal/kg/day if there is excessive weight gain [7, 22].

Post-transplant energy requirements should match those of normal children, though care needs to be taken with increased appetite in response to steroid administration. Up to 13% of transplanted chidren become obese [23, 24]. A reduction in energy intake is indicated where there is excessive weight gain and will help correct any dyslipidaemia.

### Protein

Protein intakes in CRF must provide at least 100% RNI if protein is not to become the limiting factor for growth. Inadequate protein will impact on body composition with a preponderance of fat rather than lean tissue. Adequate energy must be given to promote deposition of protein [25]. To ensure an adequate protein intake, it is advisable to use the RNI for height age if the child is below the 2nd centile for height.

In children undergoing PD, protein intake must provide at least 100% RNI plus an allowance for both replacement of transperitoneal losses and replacement of daily nitrogen losses in order to achieve positive nitrogen balance [7, 18, 22]. There are few studies describing the optimal amount of protein for children on PD and the existing data does not include all age groups. Taking this into account, and the wide variability in transperitoneal losses of protein, recommended intakes for protein for populations of children on PD may be considered generous. Routine assessment of growth, albumin and urea levels will determine the required intake for the individual child. For chronic haemodialysis, the Kidney Disease Outcomes Quality Initiative (K/DOQI) recommends the RDA for age plus an increment of 0.4 g/kg/day to achieve positive nitrogen balance [10]. This recommendation is based on work done in adults on HD who failed to maintain nitrogen balance on 1.1 g protein/kg/day [26]. Published recommended intakes for the US and the UK shown in Tables 2 and 3 are for populations and may need adjustment for the individual.

**Table 2:** US recommended dietary protein for children on maintenance dialysis [10]

	Age (yr)	RDA (g/kg/day)	Protein intake for HD (g/kg/day)	Protein intake for PD (g/kg/day)
Infants	0–0.5	2.2	2.6	2.9–3.0
	0.6–1.0	1.6	2.0	2.3–2.4
Children	1–6	1.2	1.6	1.9–2.0
	7–10	1.0	1.4	1.7–1.8
	11–14	1.0	1.4	1.7–1.8
Males	15–18	0.9	1.3	1.4–1.5
Females	15–18	0.8	1.2	1.4–1.5

**Table 3:** UK guidelines on dietary protein for children on maintenance dialysis [27]

	Age	RNI (g/kg/day)	Protein intake for HD (g/kg/day)	Protein intake for PD (g/kg/day)
Infants	0–3 mo	2.1	2.5	2.8–2.9
	4–12 mo	1.5–1.6	1.9	2.2–2.3
Children	1–3 yr	1.1	1.5	1.8–1.9
	4 yr–puberty	1.0	1.4–1.5	1.7–1.9
	Pubertal	1.0	1.3–1.4	1.6–1.8
	Post–pubertal	0.9	1.2–1.3	1.4–1.5

Post-transplant protein requirements should match those of normal children.

### Vitamins and minerals

Little is known about the requirements of children with CRF. It would be reasonable to give the RNI for vitamins, minerals and micronutrients as for normal children, with the exception of calcium, phosphate, magnesium, sodium and potassium, which may be deranged and must be determined for the individual child. UK RNIs are shown in Table 4.

**Table 4:** Micronutrient guidelines for children with CRF [28]

	Infants	Children
Thiamin (mg)	0.2–0.3	0.5–1.0
Riboflavin (mg)	0.4	0.6–1.3
Niacin (mg)	3.8	8–18
Vitamin B6 (mg)	0.2–0.7	0.7–2.0
Vitamin B12 (μg)	0.3–0.5	0.5–1.5
Folic acid (μg)*	50–500	70–1000
Vitamin C (mg)	25	25–40
Vitamin A (μg)*	350	350–700
Vitamin D (μg)*	7–8.5	–
Zinc (mg)	4.0–5.0	5.0–9.5
Copper (μg)	0.2–0.3	0.3–1.0

*Vitamin A - care should be taken not to give excessive amounts of vitamin A, as the resultant high serum levels can lead to hypercalcaemia, anaemia and hyperlipidaemia [29]. A common practice guideline is not to exceed 200% of the RNI from the diet and/or supplements.

*Vitamin D - there is no need to achieve the RNI for vitamin D, as it cannot be converted to the activated form (as which it is usually supplemented). Recent recommendations for adults are to assess 25 and 1, 25-vitamin D levels and replace as needed, but there are no equivalent recommendations for children.

*Folic acid - hyperhomocysteinaemia is an independent risk factor for cardiovascular disease [30, 31]. Additional folic acid may be given to effectively lower plasma homocysteine levels. A common practice guideline is to supplement when the glomerular filtration rate (GFR) is <40 ml/min/1.73 m^2^, but the doses used are arbitrary.

Infants: 250 μg/kg to maximum of 2.5 mg daily

Children 1–5 years: 2.5 mg daily

Children >5 years: 5 mg daily

In adults on PD, blood concentrations of some water soluble vitamins (C, B6 and folic acid) are reported to be low. This is due to a combination of inadequate intake, increased transperitoneal losses and increased needs. In children supplements of these vitamins have been given with the result that blood concentrations have met or exceeded normal values [32, 33]. Accordingly, based on these authors’ recommendations and the RNIs above, the following intakes are suggested [27]:

Vitamin C: 15 mg (infants)-60 mg (children) daily

Vitamin B6: 0.2 mg (infants)-1.5 mg (children) daily

Folate: 60 μg (infants)-400 μg (children) daily

These amounts may well be met from food, feeds and nutritional supplements so it is important to assess the dietary contribution before routinely giving medicinal supplements. Whilst adequate vitamin C needs to be given to offset dialysate losses excessive intakes should be avoided, as the resulting elevated oxalate levels may lead to cardiovascular complications [34]. If folic acid is given to lower plasma homocysteine this will more than compensate for dialysate losses of folate. The K/DOQI recommends that supplementation should be considered if dietary intake alone does not meet or exceed the dietary reference intake, if blood vitamin levels are below normal values, or if there is clinical evidence of deficiency [10].

There are no reported specific micronutrient requirements for children on HD and post transplant and 100% of the RNIs can be considered the goal for these children.

### Calcium and phosphate

Control of phosphate, calcium and parathyroid hormone (PTH) levels is necessary to prevent renal bone disease. Dietary phosphate may need to be restricted when the GFR falls below the normal range, and almost always when below 50 ml/min/1.73 m^2^. The following common practice guidelines will help maintain serum phosphate within acceptable reference ranges, although phosphate binders may also be necessary.

Infants <10 kg: <400 mg daily

Children 10–20 kg: <600 mg daily

Children 20–40 kg: <800 mg daily

Children >40 kg: <1,000 mg daily

### Iron, copper and zinc

Anaemia can be prevented by the prescription of iron supplements and erythropoietin (rhuEPO). Advice to increase dietary haem iron and reduce the inhibition of non-haem iron absorption by: phytates in wholegrains and legumes; polyphenols in tea, coffee and cocoa; calcium in dairy products; and simultaneous administration of phosphate binders and antacids should be given. It may be necesary to give intravenous iron if stores are low. Low dietary intakes of copper and zinc are reported [35] in children on PD. The K/DOQI recommends the intake of these to be monitored every 4–6 months and supplements given if necessary [10].

## Reports of spontaneous nutritional intake in children with CRF

Several studies have demonstrated decreased spontaneous intake in children with CRF. Four-day weighed dietary records from 50 children with a GFR <65 ml/min/1.73 m^2^ and 93 healthy children showed an energy intake 76–88% of the RI in CRF patients and 90%–93% in controls. Protein intake was 2.1–3.1 g/kg per day in controls and 1.6–2.7 g/kg per day in CRF patients, so that overall, the energy intake was 10% and the protein intake 33% lower in CRF patients than in healthy children [36]. Other studies have shown low energy intake (87% RNI) but high protein/energy ratio, protein (223%), carbohydrate (73%), fat (110%), polyunsaturated (55%), monounsaturated (129%) and saturated fatty acid (111%), with relative distribution of calories of 15% from proteins, 48% from carbohydrates and 37% from lipids in 15 children with moderate CRF [14]; and RIs in 82 children with CRF were <86% for energy and >161% for protein [12].

Although energy intake is low, it may be proportionate to body weight: 4-day food records from 120 children with CRF found an energy intake of 80% of RI for age, decreasing with increasing age. However, this was in the normal range when factored by body weight. The protein intake was 153% of the RDA [37].

Intake deteriorates with severity of CRF: energy intakes correlated negatively with GFR in 95 children with CRF, and fell to 85% of EAR when the GFR was <25 ml/min/1.73 m^2^ [38]. Nitrogen balance studies have been performed in 19 children on PD. Protein intake was 1.64 g/kg/day (126% of the RDA), and the calorie intake reached 75% of RDA. Nitrogen losses were 0.205 g/kg/day, and nitrogen balance was positive in three quarters of studies, correlating with nitrogen and calorie intake [39].

Intake also decreases with time: 3-day semi-quantitative dietary diaries in 51 children with a GFR <75 ml/min/1.73 m^2^ assessed over 2 years showed protein intake to decrease by 0.4 g/kg per day, and calcium by −20% RNI [40]. Energy and protein intake (energy more than protein), all anthropometric measurements and plasma proteins and AAs were low in 24 children on continuous ambulatory PD (CAPD), particularly in those <10 years of age [13].

Low intakes of calcium [35–38], zinc [35, 37, 40, 41] and vitamins [37, 40, 42] are also reported.

## The epidemiology of nutritional disturbances in renal disease, including height, weight and body composition

### Height and weight

It is during the infantile phase of growth that the most significant loss of height potential can occur, but also it is the phase during which there is the greatest potential for catch-up with nutritional intervention. Many infants with CRF are already growth-retarded by the time they are first seen in a paediatric nephrology service: a loss of HtSDS from birth of −1.68 SD (up to −5 SD/year) has been reported [43]. Approximately one-third of the reduction in height occurs during foetal life and one-third during the first 3 months, accompanied by a similar decline in head circumference [44–46]. Mean HtSDS below the lower limit of normal has been reported in most studies [43–46]. However, catch-up can occur, even on dialysis [47–49]. Poor nutritional status is associated with starting PD at a younger age [50]. Interestingly, infants who grew well continued with catch-up in early childhood [43, 47].

During the childhood phase, growth in CRF usually parallels the centiles but without catch-up [43, 46]. The North American Pediatric Renal Transplant Cooperative Study (NAPRTCS) CRF database (<20 years of age, GFR <75 ml/min/1.73 m^2^) has shown a mean HtSDS that has changed little since 1996 when it was −1.5 in 1,725 patients, to −1.4 (one third >−1.88) in 3,863 patients in 1998 and −1.4 in 4,666 patients in 2001 [51–53]. European study group data on 321 children aged 1–10 years with congenital CRF reported a mean HtSDS of −2.37. Increasing severity of CRF adversely affects growth: when the patients were divided into those with a GFR greater or less than 25 ml/min/1.73 m^2^, the HtSDS was −1.65 and −2.79, respectively [44]. Reports of growth on dialysis vary from improvement [54], to no change [55] to declining HtSDS [56], with a worsening of nutritional status in children dialysed for more than a year [50].

The pubertal phase of rapid growth is another period when loss of height potential can occur. Early studies demonstrated that puberty was delayed, with an irreversible decline in height SDS, particularly in patients on dialysis [57, 58]. However, others have reported normal pubertal progression and growth [43, 46, 59].

### Body composition

Children with CRF and short stature are significantly protein-depleted for age, although not for height: 17 patients, mean age 12.9 years had total body nitrogen (TBN) and TBN/height of 54% and 63%, respectively, when predicted from age, but 100% when predicted from height. Energy and protein intakes were 65% and 172% of RDA, respectively. This suggests that chronic energy deficiency may contribute to impaired protein deposition which, in turn, may be important in the pathogenesis of growth failure in CRF [25].

It has been suggested that the ratio of the length of trunk to limb is low in CRF, suggesting a disproportionately greater effect of disease and/or treatment on spinal growth [60] although not all have found this [61].

BIA in children starting PD showed an improvement of hydration and nutrition after 6 months, although levels remained below normal, suggesting that introduction of dialysis should not be left until malnutrition has developed [62, 63]. DEXA studies of 20 PD patients receiving the RDA for energy and a daily protein intake of 144.3% and 129.9% RI at months one and six showed an increase in BMD, bone mineral content (BMC) and FFM. However, the daily protein intake showed a negative correlation with these parameters and also plasma bicarbonate, suggesting that a high protein intake may negatively affect bone mineralization and FFM by its effect on acid-base status [64].

Disturbances in plasma and intracellular AAs have been found in CRF. Muscle isoleucine and valine levels and the valine/glycine ratio were low in children with CRF who were short but had no other signs of malnutrition (normal skinfold thickness, MAC and serum proteins) [65]. Levels have been studied in ten children on CAPD. Although plasma levels of essential AAs (EAAs) were low, only muscle intracellular leucine and valine were low, whereas both plasma and intracellular levels of some non-essential AAs were high. No correlations were found between plasma and muscle AAs and indicators of nutritional status, except muscle branched-chain AA levels with BMI [66].

## The pathological mechanisms underlying poor appetite, abnormal metabolic rate and endocrine disturbances in renal disease

Poor appetite is common in children with CRF and may in part be due to abnormal taste sensation [67]. Appetite is also affected by cytokines, and their roles in CRF, along with their effect on metabolic rate, have been extensively and excellently reviewed [68, 69]. Malnutrition may be an inappropriate term in CRF because it infers that dietary replacement would be curative (which is not always the case in CRF), and the term cachexia, which implies replacement of muscle with fat and declining plasma proteins, may be more appropriate [68, 69]. Cachexia may result not only from anorexia but also from acidosis and inflammation (which are common in CRF), which cause elevated levels of cytokines such as leptin, TNF-α, IL-1 and IL-6. These act through the hypothalamus to affect appetite and metabolic rate and maintain the constancy of fat stores.

Leptin is produced by adipocytes and is probably the most important cytokine involved in this process. When body fat falls, levels of leptin decline and the brain responds by increasing appetite and metabolic efficiency; in contrast, when leptin levels rise, food intake is decreased and metabolic rate increases. However, leptin is excreted by the kidney and not cleared by dialysis, so levels can be paradoxically high in malnourished patients, contributing further to decreased food intake and increased metabolic rate. Serum leptin levels were >95th percentile in 45% of 134 children with varying severity of CRF, and correlated positively with their percentage body fat and GFR and negatively with spontaneous energy intake [70]. Leptin levels also correlate with CRP, a marker of inflammation, and with insulin resistance [67, 69]. Leptin signalling in the brain occurs through the hypothalamic melanocortin receptors, and may offer a new area for therapeutic intervention in the cachexia of CRF. The part played by the short-term regulators of satiety, such as ghrelin (which stimulates appetite), is not yet fully understood [68, 69].

Abnormalities of the GH/IGF-1 axis occur in CRF. Whether they are primarily due to CRF itself or secondary to malnutrition is controversial [71, 72]. GH levels are normal to high and IGF-1 levels are low in both CRF and malnutrition [73]. IGF-1 decreases according to nutritional status in children on HD [71]. Leptin levels correlate positively with IGF-1 and negatively with GH in children with starvation [73]. However, in CRF this relationship is disrupted: in 17 children on HD with energy and protein intakes of 40–70 kcal/kg per day and 1–1.54 g/kg per day, respectively, who had reduced anthropometric measurements, although IGF-I levels were low, leptin levels were high [74].

## Evidence for the benefit of dietetic input, dietary supplementation, nasogastric and gastrostomy feeds and intradialytic feeding

There are very few controlled trials, so recommendations are based on the evidence available. Some, but not all, reports have been able to establish a relationship between nutritional intake and growth. Growth velocity correlated positively with energy intake in 17 children with CRF [75], and with energy but not protein intake in 15 children on CAPD [76]. Growth velocity was inversely correlated with dietary protein intake and positively correlated with caloric intake both before the initiation of rhGH therapy and after the first year of treatment in 31 children on dialysis [77]. The advantage of input from a dietitian has been specifically demonstrated in two studies [11, 40], but it is likely that in all studies dietetic input was necessary.

### Dietary supplements

Because of the importance of nutrition in the first two years of life it might be expected that enteral nutrition would be most effective at this age. Most studies have therefore concentrated on this age group, and there is evidence to show that nutritional supplementation is of benefit. Eight studies have demonstrated an improvement in growth [21, 47, 78–83], three showed an initial decline followed by stabilisation [84–86], three showed no effect on growth [11, 87, 88] and one showed a decline in HtSDS [89]. All but one study [86] used feeds administered by nasogastric or gastrostomy tubes and aim for at least the EAR for energy and RNI for protein, with a protein supplement for dialysis.

An early study of nasogastric feeding in 14 children weighing <10 kg demonstrated a benefit on HtSDS and WtSDS in 11 [78]. Twenty-six children aged <2 years with a GFR <26 ml/min/1.73 m^2^ were treated with a whey-based infant formula (supplemented with fat and/or carbohydrate) which provided 100% of the RNI for protein for Ht age and 100% of the EAR for energy for chronological age. HtSDS increased from −2.9 to −2.1 over 2 years [21]. A similar feed resulted in improvement in HtSDS from −2.34 at 6 months of age to −1.93 at 2 years in 24 infants with a GFR <20 ml/min/1.73 m^2^, and from −2.17 to −1.24 in 13 infants on dialysis over a similar time-frame, although a protein supplement for dialysis was included [47]. A further study from the same centre showed an increase in HtSDS from −1.8 to −0.8 at 2 years in 20 infants on PD [79]. Three studies, each reporting the results of nasogastric feeding in three young children with CRF, have shown benefit in eight out of the nine children [80–82]. It is important not to restrict the salt and water content of the diet in the salt-wasting polyuric infant: a feed providing just over the RDA for calories and protein diluted to 0.3–0.5 kcal/ml with additional 2–4 mEq of sodium/100 ml in 24 infants resulted in improvement of HtSDS by 1.37 SD at 1 year and 1.82 SD at 2 years [83].

Three studies have shown an initial decline followed by normalisation of growth. Twelve infants with CRF dropped to a HtSDS of −2 by 12 months of age and then stabilised at that level [84]. Decline in HtSDS was arrested in eight children <2.5 years of age after starting gastrostomy feeds [85] and stabilised after 3 months in the only study using supplementation without nasogastric or gastrostomy feeds [86]. In three studies, enteral feeds made no impact on growth: there was no change in infants with CRF [87], HD [88] or PD [11]. In one study, a decline in HtSDS occurred in 82 children <2 years of age at the start of dialysis [89].

Whether supplemental feeds benefit children over 2 years of age has been challenged [90]. Six studies have included older children [21, 80, 81, 85, 89, 91]. Of these there was an improvement in growth in three [80, 81, 91]. Dietary advice and supplements of glucose polymer and vitamins (as Ketovite) were given to 65 children aged 2–16 years with a GFR <75 ml/min/1.73 m^2^ if their EAR and RNI, respectively, fell below 80% as assessed by annual 3-day dietary diaries. Mean HtSDS was maintained in those with a GFR of 25–75 and significantly increased in children with a GFR <25 ml/min/1.73 m^2^. There was an increase in HtSDS and/or BMI SDS in all the patients on supplements, and change in energy intake correlated with change in HtSDS in those with a GFR <25 ml/min/1.73 m^2^ [91]. Three children with a GFR of 20–25 ml/min/1.73 m^2^ fed overnight by nasogastric tube for 11–16 months with increasing amounts until weight gain occurred improved their HtSDS [81], as did three children with CRF over the course of a year [80]. Two studies have shown no change in HtSDS. Nine children aged 2–5 years, treated with a whole protein enteral feed supplemented with fat and/or carbohydrate showed no change in HtSDS (−2.3 to −2.0) [21], as did seven children in given gastrostomy feeds [85]. One study has demonstrated an ongoing decline in HtSDS in children aged 2–5 years at the start of dialysis, 14 of whom were given supplements and 20 not [89].

Complications of gastrostomy are uncommon, but include gastro-colic fistulae, paraoesophageal herniae and, in children on PD, post-surgical peritonitis and an increased risk of exit site infection and dialysis catheter removal from infection, a risk that might be reduced if open rather than percutaneous surgery is used [92, 93]. After removal the track usually closes spontaneously [94]. There have been concerns that enteral tube feeding precludes the development of normal feeding behaviour [95]. However, other studies have shown that, despite long term nasogastric or gastrostomy feeding, oral feeding is resumed in the majority of children after successful transplantation [21, 96]. Positive reinforcement at feeding times using behavioural therapy techniques allowed five infants who had PD and nasogastric tube feeding initiated in the first month of life, and who showed persistent food refusal, to convert to oral feeding [97]. Our impression is that spontaneous oral intake increases with long-term tube feeding; indeed, over a period of 31 months energy intake from the feed did not increase, implying that oral intake had improved over this time to support the demonstrated growth [21]. Reports of the use of Nissen fundoplication are principally from one group [21, 47, 54, 79, 88, 92], making assessment of its effect difficult, although results of growth from this centre are good.

### Essential aminoacid (EAA) supplements

Serum EAAs, carnitine and total protein levels have been demonstrated to be low in CRF, particularly in patients on PD [98]. It has been suggested, therefore, that a low protein diet supplemented with EAAs might benefit growth by ensuring adequate AA intake without protein toxicity. However, results have been inconclusive. No improvement in growth was seen in seven children with severe CRF who were given half the protein RDA for height age as EAAs for 6–8 months [99]. Ten children with CRF managed for 3 years using a strict low protein diet supplemented by a mixture of the keto and amino forms of the EAAs and histidine showed a significant increase in height and weight velocity [100]. HtSDS improved from −1.93 to −1.37 over 30 months in 20 patients with a GFR <50 ml/min/1.73 m^2^ on a diet of 0.6 g/kg of protein supplemented with ketoacids [101]. Ten children on HD given AA supplementation (0.25 g/kg body weight i.v.) with and without carnitine (25 mg/kg body weight i.v.) had no overall improvement in AA levels [102].

### Amino acid-containing peritoneal dialysis solutions

Excessive glucose absorption and dialysate AA and protein losses contribute to malnutrition in children on PD. It has been suggested, therefore, that using an AA dialysate might both decrease glucose load and replace AA losses. AAs are absorbed in proportion to the concentration difference between dialysate and plasma; after a 1% AA exchange in seven children on CAPD, the rise in plasma levels of AAs correlated with the ratio of the amount of AA in the bag to the basal plasma concentration. The amount of AA absorbed was 66% after 1 h, and 86% after 4 h and 6 h [103, 104].

However, there is no evidence for any long-term nutritional benefit: eight children on CAPD who had a first morning exchange of 1% AA dialysate instead of dextrose for 12–18 months had no improvement in any plasma or anthropometric parameter of nutrition; plasma urea increased. Plasma EAAs, which had been low, improved but the intracellular pool of free AAs, measured in polymorphonuclear leucocytes did not improve [105]. Two randomised prospective cross-over studies of 3 months AA or dextrose dialysate for three months have been performed, both in seven growth-retarded children either on CAPD [106] or continuous cycling PD (CCPD) [107]. In the children on CAPD there was no nutritional benefit from the AA dialysis [106]. The children on CCPD received dextrose dialysate overnight, plus a single daytime dwell of either AA dialysate or dextrose dialysate. Appetite, calorie and protein intake improved and total body nitrogen increased in half the children during AA dialysis. However, total plasma protein and albumin did not change and fasting AAs after 3 months of AA dialysis were comparable to baseline; plasma urea concentrations were higher [107]. High plasma urea may be due to inadequate protein synthesis in the absence of glucose. Ten children underwent overnight CCPD using a 3:1 ratio of glucose to AA solutions simultaneously during the night. Glucose absorption was 33.7% and AA absorption was 55.2% of the infused amount, and although plasma AA levels were high for the entire ambulatory PD (APD) treatment the plasma urea levels did not increase suggesting that the AAs were being used for protein synthesis with this regimen [108].

Disadvantages compared with glucose include cost, and reports of fluid removal are variable. However, equal amounts of urea and creatinine are removed, normoglycaemia is maintained and there are no reported adverse clinical or biochemical effects, other than a slight increase in plasma urea [106, 107, 109].

### Intradialytic parenteral nutrition (IDPN)

There are only four studies of IDPN during HD in children so it is difficult to draw conclusions about its effectiveness. Losses of AAs occur into the dialysate during HD and depend on their plasma concentrations and molecular weights. AAs were added to the dialysate of three children in increasing concentrations. Plasma nonessential AAs were not affected, but EAAs improved [110]. Four malnourished children on HD were given IDPN as AAs (8.5% solution), glucose (10% to 15% dextrose), and 20% fat emulsion at every dialysis session (three times a week) for 7–12 weeks. Oral intake improved and, although weight did not improve during treatment, it did so subsequently. Albumin did not change [111]. The weights of three children improved after 6 weeks of IDPN; again, albumin did not improve [112]. Nine patients who on HD had a >10% weight loss and were <90th percentile of ideal body weight received thrice weekly IDPN. In six, BMI increased in the first 5 months and PCR increased, whereas serum albumin did not change; those who did not gain weight were considered to have psychosocial causes for their malnutrition [113].

### Nutritional causes of poor growth not related to energy and protein

#### Sodium

Requirements for sodium vary according to the type of renal disease. Congenital structural abnormalities often result in an obligatory urinary loss of salt and water. Such children may become chronically salt- and water-depleted and need sodium supplementation and free access to water as salt wasting impairs growth [83]. On the other hand, children with glomerular disease need to restrict their sodium intake. Young children on PD may need sodium supplementation as considerable sodium losses can occur in dialysate.

#### Acidosis

Acidosis is associated with a catabolic state, suggesting that acidosis-related protein wasting could contribute to growth retardation [114, 115]. Correction of acidosis improves serum albumin, catabolic rate and growth [83, 116, 117].

#### Anaemia

Anaemia is a well recognised cause of poor appetite. However, studies that include blinded, placebo-controlled trials have found that despite subjective increases in appetite, there were no consistent improvements in dietary intake or anthropometric measures observed during rhuEPO treatment [118–122].

#### Vitamin D

The dose of calcitriol prescribed to control hyperparathyroidism must be balanced against its potential to depress the activity of chondrocytes causing adynamic bone disease. Large doses impair growth, even if intermittent [123, 124], but the frequency of administration does not affect growth if small doses are used [125, 126].

### Growth hormone

There may be cases when, despite at least 6 months of adequate nutrition, growth continues to be poor. GH may be offered in these circumstances.

## The effect of dialysis adequacy on nutrition

Several studies have looked to see whether increasing dialysis dose benefits appetite, protein intake, nutrition and growth. Twenty-one children on CCPD showed an improvement in HtSDS when aiming for a Kt/V of >2 and a creatinine clearance of >60 l/week/1.73 m^2^ compared with 1.7 and 40 l/week/1.73 m^2^ [127].

However, in some, but not all studies, there would appear to be a ceiling above which no further benefit occurs. The nPCR and serum albumin were assessed according to Kt/V in 15 patients on HD. Serum albumin levels were normal. The nPCR was lowest in patients with a Kt/V <1.3, but increasing over 1.6 did not improve nPCR further, suggesting that although adequate dialysis needs to be achieved in order to ensure good protein intake, high dialysis doses are of no further benefit [128]. However, in 12 children taking 90.6% and 155.9% of their requirements for energy and protein, respectively, and receiving HD with a Kt/V of 2.00 and a urea reduction ratio of 84.7%, there was an improvement in HtSDS of +0.31 SD/year and pubertal growth was normal, suggesting that for HD, increasing dialysis dose does improve growth [129].

In PD, but not HD, there was an inverse relationship between albumin level and Kt/V, suggesting that increasing PD dose may reach a point of no further benefit due to increasing albumin losses in PD fluid [130]. PD causes an influx of glucose which can contribute to obesity. High transporter status was associated negatively with HtSDS and positively with BMI SDS in 51 children on PD. Large dialysate volumes also affected BMISDS [131].

Improving dialysis dose can be achieved by the addition of an icodextrin daytime dwell to overnight PD. A cross-over study in eight children of overnight PD with or without addition of a daytime dwell with 1,100 ml/m^2^ icodextrin for a week showed an improvement in weekly dialysis creatinine clearance from 35 to 65 l/week/1.73 m^2^ and Kt/V from 1.99 to 2.54. However, protein and calorie intake did not improve and peritoneal albumin loss and serum albumin did not change, but there was increased loss of AAs, although plasma AA levels did not change [132].

Residual renal function (RRF) has an important positive effect on clearance and growth. Mean HtSDS improved from −1.78 to −1.64 over a year of PD in 12 patients with RRF, but declined from −1.37 to −1.90 in 12 patients without. Weekly Kt/V was not different, and only the native kidney Kt/V and creatinine clearance correlated with growth, suggesting that clearance obtained by PD cannot be equated with that obtained by native kidneys [133]. Eleven of 20 patients on PD with a minimum total Kt/V of 2.1, daily protein intake of 3.25 g/kg/day and HtSDS of −2.3 improved their HtSDS by 0.55, while in nine it declined by −0.50. Variables affecting growth were nitrogen balance and residual Kt/V [134].

## The role of nutrition in the outcome of children with CRF

Malnutrition is associated with increased mortality. The association with plasma albumin levels has already been discussed [12]. Of 2,306 children, those with a HtSDS less than −2.5 at the start of dialysis had a twofold higher risk of death [135]. In 1,949 children with ESRF, each decrease in height by 1 SD was associated with a 14% increase in risk for death, and there was a U-shaped association between BMI and death [4]. Part of this may be due to an increased risk of infection in malnourished patients [136].

*In conclusion*, commencement of careful nutritional support early in the course of disease may improve not only growth but also mortality in children of all ages with CRF.

## Multiple choice questions

(Answers appear following the reference list)

For each question answer *true* or *false*:

1. During the phases of growth
  - Rate of growth is highest during prenatal life
  - 50% of final height is achieved by the age of 2 years
  - The infantile phase of growth is principally dependent on growth hormone
  - Growth rate stays the same throughout the childhood phase of growth
  - A pubertal growth spurt can occur without the development of secondary sexual characteristics
2. Assessment of nutritional status in CRF and on dialysis
  - It may be more appropriate to express measures of growth according to height age rather than chronological age
  - The height standard deviation score (HtSDS) is the number of standard deviations from the mean for a normal population of the same age and sex
  - The BMI is the Ht/Wt^2^ and indicates the proportion of fat mass to fat free mass
  - Low serum albumin is always an indication of malnutrition
  - Peritonealprotein losses in dialysate are twofold greater in relation to body surface area in infants than in those >50 kg in weight
3. Nutritional requirements
  - The estimated protein requirement for a normal healthy 30-week-old girl weighing 6.0 kg (0.4th centile) and 59.0 cm in length (<0.4th centile) is >2.1 g/kg/day
  - Her estimated energy requirement is 150 cal/kg/day
  - The prescribed dietary protein intake for the same child on PD would be 2.8–3.0 g/kg/day
  - Children with CRF or on dialysis need a calorie intake that exceeds the EAR for height age
  - Supplements of vitamin A are necessary in children on dialysis
4. Appetite and metabolic rate
  - Abnormal taste sensation can occur in CRF and on dialysis
  - Leptin is produced by adipocytes and levels are high in CRF and on dialysis
  - High leptin levels cause an increase in food intake and metabolic rate
  - GH levels are normal or high and IGF-1 levels are low in CRF
  - GH levels are normal or high and IGF-1 levels are low in malnutrition
5. Dietary supplementation
  - Salt restriction is important in all children with CRF
  - Nutritional supplementation has not been shown to benefit children over two years of age
  - Increasing dialysis dose in PD may increase peritoneal dialysate protein losses and contribute to obesity
  - Sodium supplementation may be necessary in children on PD
  - Gastrostomy placement is preferable before PD commences
